# The use of tissue plasminogen activator as continuous infusion into an arteriovenous hemodialysis access in the hemodialysis unit: a case series

**DOI:** 10.1186/s40697-015-0035-z

**Published:** 2015-01-30

**Authors:** Elene van der Merwe, Rick Luscombe, Mercedeh Kiaii

**Affiliations:** Department of Medicine, Division of Nephrology, St Paul’s Hospital, Vancouver, BC Canada; University of British Columbia, Vancouver, BC Canada; St Paul’s Hospital, Providence Building, 1081 Burrard Street, Vancouver, BC V6Z1Y6 Canada

**Keywords:** Tissue plasminogen activator, Arteriovenous dialysis fistula, Continuous infusion, Dialysis unit

## Abstract

**Background:**

Thrombolytics such as tissue plasminogen activator are often used by interventional radiologists in the radiology suite to salvage thrombosed hemodialysis arteriovenous fistulae. Thrombolytics are also commonly used in dialysis facilities as a dialysis catheter lock solution or as an infusion into a dialysis catheter when dysfunctional. However, the use of tissue plasminogen activator as a continuous infusion into an arteriovenous fistula in the dialysis facility to treat clot burden is not commonly done.

**Objective:**

The aim of our case series is to demonstrate the successful use of tissue plasminogen activator to decrease clot burden in an arteriovenous fistula in the dialysis unit.

**Design:**

Observational case series.

**Setting:**

An outpatient dialysis facility in a tertiary care hospital.

**Patients:**

Three non- consecutive patients were diagnosed with an acute thrombosed arteriovenous fistula either on physical exam or by imaging.

**Measurements:**

A thrombosed fistula as well as successful resolution of clot was illustrated by either physical exam, fistulogram or ultrasound. A functioning fistula demonstrated successful continuation of dialysis and adequate access flow measurements in follow up.

**Method:**

This is a case series that describes the technique of using an infusion of tissue plasminogen activator into an arteriovenous fistula on the dialysis unit by vascular access nurses to treat or diminish clot burden.

**Results:**

We have described three separate cases where treatments with tissue plasminogen activator infusions on the dialysis unit resulted in objective evidence of decrease in clot burden on ultrasound or fistulogram.

**Limitations:**

The small numbers of our case series requires that the results need to be verified in a larger study. Inclusion and exclusion criteria need to be defined before widespread application of this technique.

**Conclusions:**

To our knowledge this small case series is the first to describe the procedure whereby low dose tissue plasminogen activator is directly infused into the fistula by the vascular access nurse in the dialysis unit during dialysis and not in the interventional suite. This provides additional information to the existing literature that there is an alternative option for dialysis units to diminish clot burden until a more permanent solution is established through angioplasty.

## Abrégé

**Contexte:**

Les agents thrombolytiques, dont l’activateur tissulaire du plasminogène, sont fréquemment utilisés en salle de radiologie par les radiologistes interventionnels dans le but de rétablir l’accès des fistules artérioveineuses pour hémodialyse qui sont thrombosées. Les agents thrombolytiques sont aussi souvent utilisés sur les services de dialyse comme solution de verrouillage de cathéter de dialyse, ou en perfusion dans un cathéter de dialyse non perméable. Par contre, l’utilisation d’un activateur tissulaire du plasminogène en perfusion continue dans une fistule artérioveineuse, comme traitement thrombolytique, est inusité sur un service de dialyse.

**Objectif:**

Le but de notre série de cas est de démontrer qu’il est possible d’utiliser, avec succès, un activateur tissulaire du plasminogène pour réduire la thrombose de la fistule artérioveineuse, sur une unité de dialyse.

**Type d’étude:**

Étude de série de cas axée sur l’observation.

**Contexte:**

Un service de dialyse ambulatoire d’un hôpital de soins tertiaires.

**Patients:**

Trois patients non consécutifs ont reçu un diagnostic de thrombose aiguë de fistule artérioveineuse, lors d’un examen physique ou par imagerie.

**Méthode:**

Cette série de cas fait la description de la méthode utilisée par les infirmières responsables de l’accès vasculaire, sur un service de dialyse, pour perfuser un activateur tissulaire du plasminogène à l’intérieur de la fistule artérioveineuse, dans le but de traiter ou de réduire la thrombose.

**Résultats:**

Nous décrivons trois cas distincts pour lesquels le traitement utilisant des perfusions avec un activateur tissulaire du plasminogène, sur le service de dialyse, a permis de documenter objectivement une diminution de la thrombose lors d’une échographie ou d’une fistulographie.

**Limites de l’étude:**

Le nombre limité de cas observés dans notre série de cas fait en sorte que les résultats doivent être vérifiés lors d’une étude plus vaste. Les critères d’inclusion et d’exclusion doivent être définis avant une plus grande application de cette méthode.

**Conclusions:**

Autant que nous sachions, cette petite série de cas est la première à décrire la procédure dans laquelle de faibles doses d’activateur tissulaire du plasminogène sont perfusées directement dans la fistule par l’infirmière responsable de l’accès vasculaire d’un service de dialyse, et non pas en salle de radiologie interventionnelle. Les données de cette étude viennent s’ajouter à celles de la littérature du domaine stipulant qu’il existe une solution pour réduire la thrombose sur les services mêmes de dialyse, et dans l’attente d’une méthode plus durable par angioplastie.

## What is known before

Thrombolytics infusion into a thrombosed arteriovenous fistula by interventional radiologists in the radiology suite is an established salvage technique.

## What this adds

This is the first description of successful use of a continuous infusion of tissue plasminogen activator into an arteriovenous fistula in the outpatient dialysis setting to decrease clot burden.

## Background

The vascular access (VA) of choice in most hemodialysis (HD) patients is the native arteriovenous fistula followed by the prosthetic arteriovenous access often referred to as the arteriovenous graft.

The most common cause of VA loss is access thrombosis which can result from various causes, the predominant one being outflow vein stenosis. Most hemodialysis programs have implemented various monitoring and interventional protocols to help maintain the long term patency and function of a dialysis patient’s VA.

Various techniques have been explored to salvage arteriovenous fistulae (AVF) and grafts. The first report of the successful use of thrombolysis of a thrombosed arteriovenous hemodialysis fistula was reported in 1985 [1]. Some of the techniques used include pharmacomechanical thrombolysis [2], mechanical thrombectomy [3] and recombinant tissue plasminogen activator with the lyse-and-wait technique [4,5]. Pulse-spray pharmacomechanical thrombolysis (PSPMT) with urokinase has been very popular until it has been withdrawn from the market in the late 1990s [6,7]. Salvage of fistulas is of utmost importance to the longevity of chronic dialysis patients that relies completely on their access site for continuation of treatment.

Reports using thrombolytics such as tissue plasminogen activator (tPA) in AV fistulae describe this procedure being used in a radiology suite [8-10] and administered by interventional radiologist usually in doses of greater than 4 mg of tPA.

Our case study series is unique in that it describes the use of tPA infused directly into HD AVF as continuous infusion during dialysis in an outpatient hemodialysis unit by hemodialysis nurses with clinical expertise in vascular access.

## Methods

Three patients were identified by either physical exam or image study to have a thrombosed or partially thrombosed arteriovenous fistula estimated to have happened in the past 48 hours. Patients who had a recent bleeding event (eg. gastrointestinal, intracranial) were excluded. Written consent was obtained from all participants prior to infusion of tPA in the dialysis unit.

Alteplase (tPA) was prepared to a final concentration of 1 mg/ml by withdrawing 2.2 ml of sterile water and injecting the water into a 2 mg vial containing alteplase in a powder form. The reconstituted solution should be used within 8 hours and stored at 3 to 30°.

**Figure 1:**
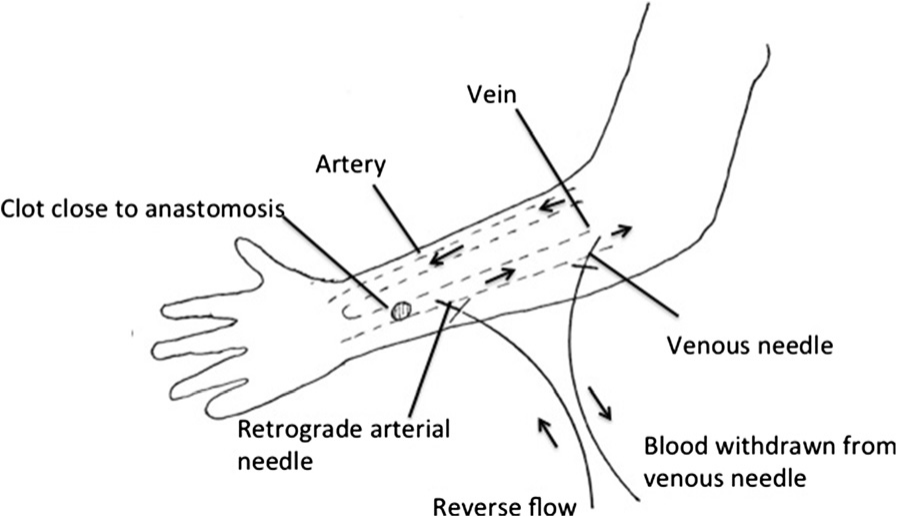
**Schematic illustration of needle placement when clot burden is close to the anastomosis site with dialysis circuit in reverse flow.**

An ultrasound or fistulogram were reviewed and the location of the clot was identified. If the clot was too close to the anastamosis site (Figure 1), the arterial needle was placed retrograde into the fistula (pointing towards the anastamosis), and the dialysis circuit was put into reverse flow (blood is infused back through the arterial needle and withdrawn from the venous needle).

**Figure 2:**
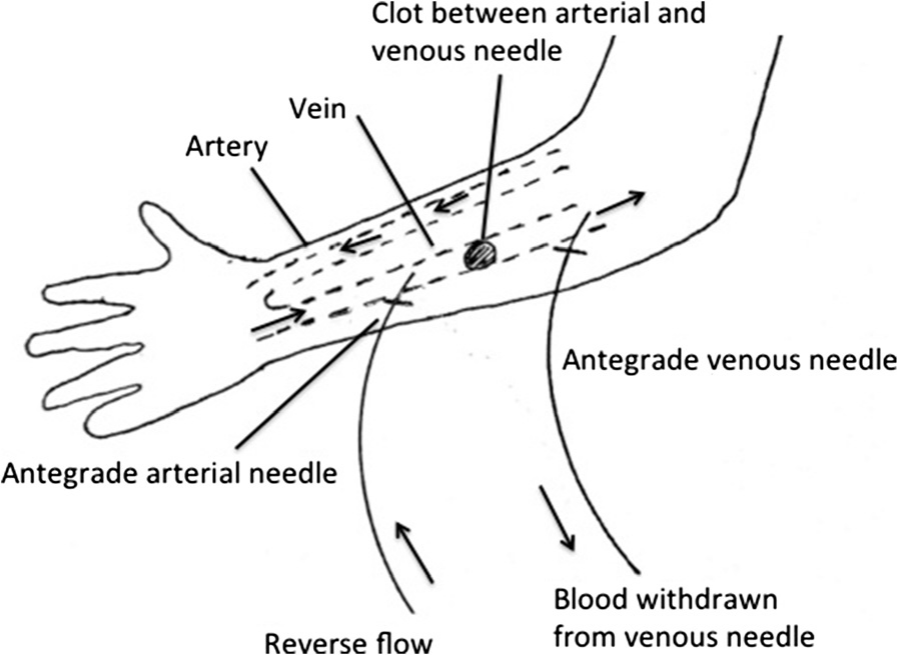
**Schematic illustration of needle placement when clot burden is located between the arterial and venous needle with the circuit in reverse flow.**

Similarly, if the clot was between the arterial needle insertion site and the venous needle insertion site (Figure 2), the flow will be reversed again but this time both needles will be placed antegrade, away from the anastomosis.

However, if the clot was beyond the venous needle site in the venous outflow towards to the heart, needles were placed antegrade and the patient was dialyzed in the conventional circuit where blood is withdrawn from the arterial needle and returned via the venous needle. Once the needles were in place, tPA was infused via a Y-connector attached to the dialysis needle. A dose of 2 mg of tPA in 50 ml of normal saline was infused over 30 minutes if the arterial needle was used as the venous return, to reduce recirculation time. If the venous needle was used as the venous return, tPA was infused over 60 minutes. Once the tPA is infused, the circuit was returned back to conventional mode. The needle size was not altered from the patient’s usual needle size used for cannulation of their fistula. The extra-corporeal heparin dose used during dialysis was not changed. We defined the tPA infusion procedure as being successful based on changes in the physical assessment of the AVF (softness, presence of bruit and thrill) and on resolution of clot burden assessed by either ultrasound, fistulogram or absence of clot aspiration on needling the fistula.

### Case series

St Paul’s Hospital is a tertiary care facility that services the needs of approximately 225 outpatient hemodialysis patients. A dedicated group of individuals manage the vascular access requirements of the dialysis patients at St Paul’s hospital. This Vascular Access team includes a nephrologist and two vascular access nurses. The vascular access nurses have been trained to use ultrasound in combination with physical evaluation to assess fistula patency. Interventional procedures are performed by a group of interventional radiologists and vascular surgeons. Three non-consecutive patients were identified with thrombosis of their AV access sites.

### Case 1

**Figure 3:**
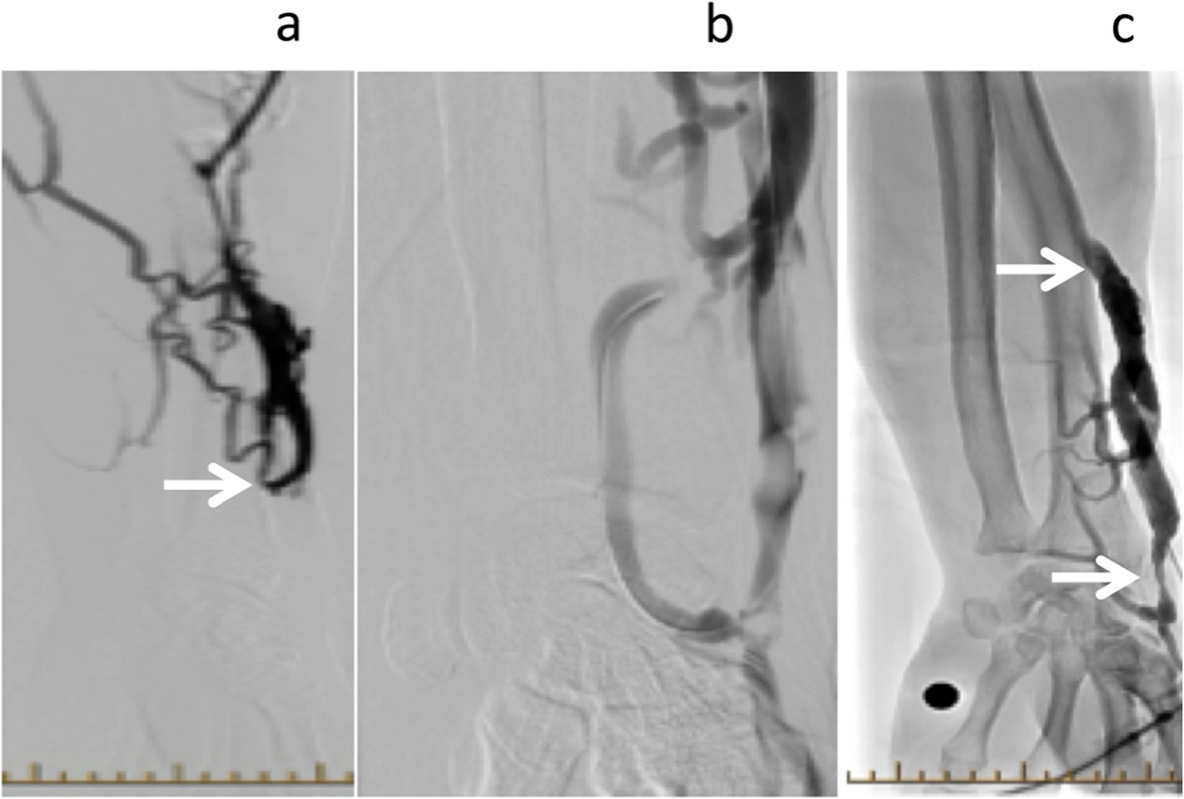
**Fistulogram images of radio-cephalic fistula. a**: Fistulogram unable to demonstrate anastomosis with a clotted radio-cephalic fistula and no flow of contrast material past the point of the stenosis (arrow indicating stenosis). **b**: Post pharmacomechanical thrombolysis in radiology suite demonstrating poor flow through the same fistula with heavy clot burden. **c**: Fistulogram one week later with repeat angioplasty of existing stenosis, after hemodialysis tPA infusion. No clot was demonstrated. Residual stenosis indicated by arrows.

A 66 year old man presented to our outpatient hemodialysis unit with a thrombosed left radio-cephalic AVF. A month prior he had an access flow of 1190 ml/min. He underwent an urgent fistulogram (Figure 3a) that demonstrated a completely occluded fistula. An angioplasty was performed. Partial recovery of flow was achieved but heavy residual clot burden was seen (Figure 3b). Once back in the hemodialysis unit, the fistula was cannulated and tPA was infused as per protocol. Following the tPA infusion the patient was dialyzed successfully. A fistulogram one week later demonstrated no clot but there was evidence of residual stenosis still present (Figure 3c). This was successfully treated with repeat angioplasty. Access flow evaluation a month after angioplasty was 1420 ml/min. He was on ASA 81 mg once daily during this time period. He has since passed away from a malignancy related complication.

### Case 2

**Figure 4:**
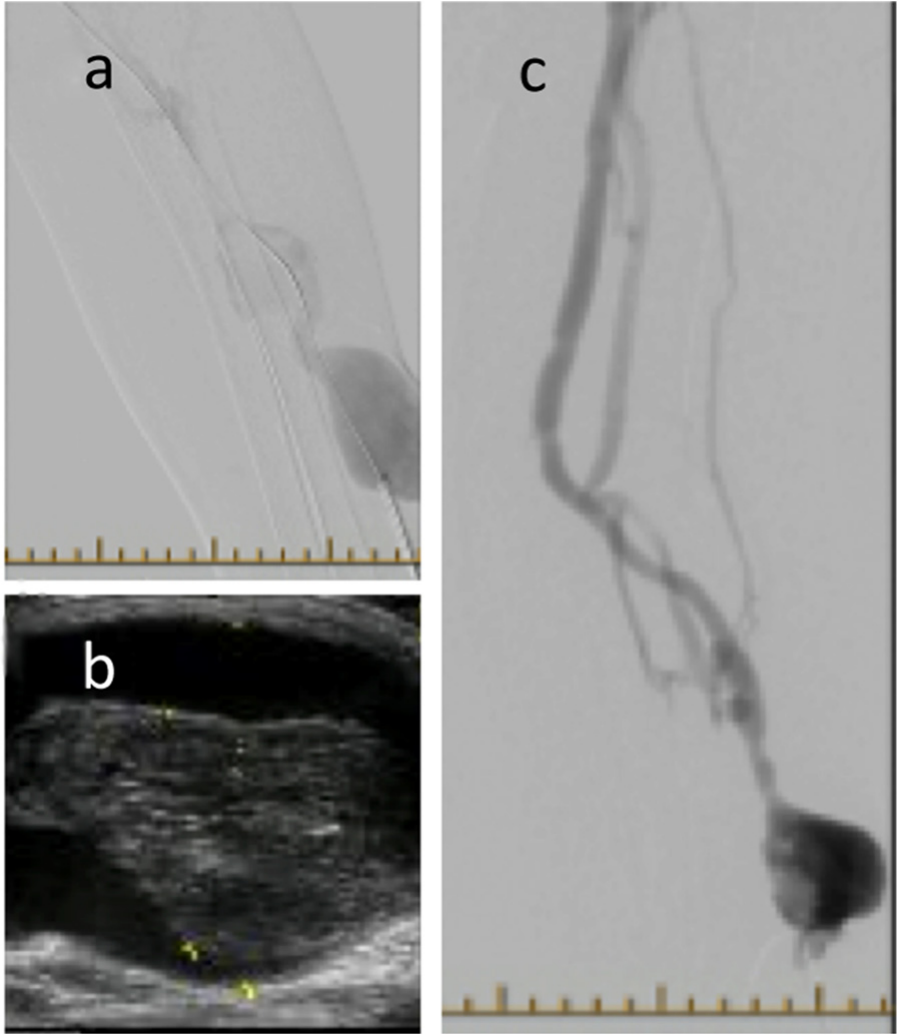
**Fistulogram and ultrasound images of radio-cephalic fistula. a**: Fistulogram after attempted angioplasty and before tPA infusion. Clot is seen in the venous aneurysm. **b**: Ultrasound demonstrates a persistent large clot in the venous aneurysm. **c**: Fistulogram of venous aneurysm one week after tPA infusion, demonstrating significant decrease in clot burden.

A 66 year female due for her regular dialysis, was found to have no thrill on careful physical examination of her left radio-cephalic AVF and dark clotted blood was aspirated on cannulation. Her access flow was adequate a month prior to presentation at 1020 ml/min. She underwent an urgent fistulogram and angioplasty but the procedure was deemed unsuccessful since adequate flow was not established and there was evidence of large residual clot burden (Figure 4a).

She returned the following day for insertion of a dialysis catheter. On clinical assessment of the fistula, a faint thrill was present. It was felt she could have a salvageable fistula and likely had vessel spasm post angioplasty, which can make it difficult to judge the viability of fistulae immediately post procedure. An ultrasound (Figure 4b) showed a large clot in the venous aneurysm. She had tPA infused as per our protocol on three consecutive days. Significant reduction of clot burden was demonstrated a week later with a repeat fistulogram (Figure 4c). The fistula was needled and successful dialysis followed. No clot burden was demonstrated on repeat ultrasound in vascular access clinic. A follow up access flow measurement a month later was 940 ml/min. She has not required any further interventions to date. She was not on any oral anticoagulants.

### Case 3

**Figure 5:**
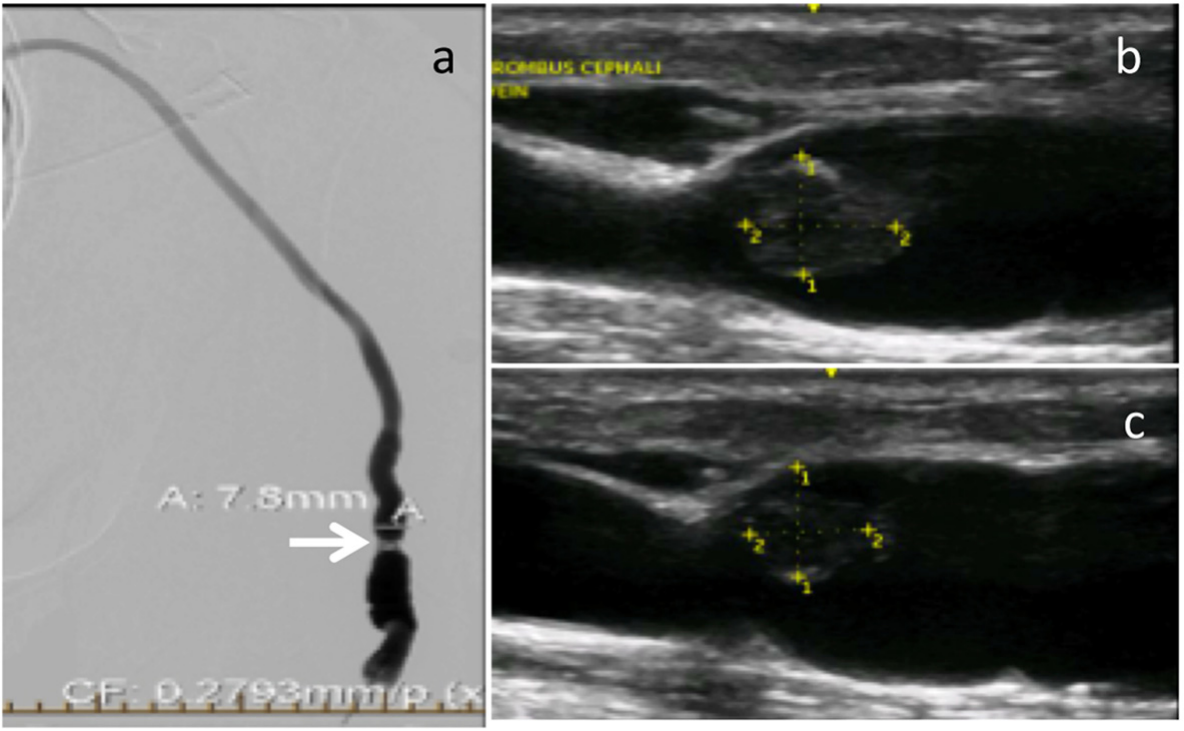
**Fistulogram and ultrasound images of brachio-cephalic fistula. a**: Fistulogram of Brachio-Cephalic AVF demonstrating a stenosis mid humerus (arrow). **b**: Doppler flow ultrasound of the AVF with large residual clot. **c**: Repeat ultrasound after tPA infusions resulted in decrease of clot burden.

A 73 year old female from our Kidney Care Clinic, (pre-dialysis, CKD stage 5), with a mature brachiocephalic AVF was assessed at 6 months post fistula creation. On physical examination of her AVF, she was found to have changes on palpation and auscultation of her AVF suggestive of a venous outflow stenosis. A fistulogram confirmed a venous outflow stenosis. Successful angioplasty of the stenosis followed. A few months later she complained of persistent pain at the fistula site. Physical exam demonstrated a hard marble like structure within the AVF. Dialysis was attempted unsuccessfully. A repeat fistulogram indicated persistent stenosis mid-humerus (Figure 5a) and was treated with angioplasty. A follow up ultrasound a week later showed residual clot (Figure 5b). She underwent four tPA infusions in the dialysis unit, on four separate days. Decrease of clot burden was evident on a repeat ultrasound (Figure 5c). On physical assessment, the marble like structure was absent and persistent pain resolved. Successful dialysis treatments were initiated. First access flow was 970 ml/min after initiation of dialysis. She underwent a repeat angioplasty for a stenosis 2 months later.

## Results

Three patients were identified to have a thrombosed or partially thrombosed AVF (2 females and 1 male). Two were brachio-cepahlic and one was a radio-cephalic fistula. All three patients demonstrated decreased clot burden after tPA infusions evidenced by fistulogram, clinical exam or ultrasound. Two of the three patients required repeat fistulogram and angioplasty after resolution of thrombosis following TPA infusions. All three patient resume successful dialysis after TPA. Adequate access flows of 1420, 940 and 970 ml/min for patient 1, 2 and 3 respectively were demonstrated in follow up**.** No obvious clinical complications were experienced during tPA infusions on the dialysis unit in any of the cases. There was no change in hemoglobin pre and post procedure in any of the patients. Bleeding times did not change in any of the 3 patients at the end of dialysis.

## Discussion

It is not unusual for dialysis nurses to aspirate clot from aneurysmal or dysfunctional AVF on cannulation. Clots in the AVF can form as a result of turbulent blood flow in aneurysmal areas of the AVF or due to trauma from previous needling. It is also possible to have residual clot in a fistula post procedure, such as angioplasty. Three patients were identified of having residual clot burden either by physical examination or demonstration by ultrasound. There is currently no guidelines or protocols in place for dialysis nurses to address this issue. The concern is that residual clot burden may lead to complete thrombosis of the arteriovenous access or result in distal embolization of the clot.

To our knowledge this small case series is the first to describe the procedure whereby low dose tPA is directly infused into the AVF by the vascular access nurse in the dialysis unit during dialysis and not in the interventional suite. This provides additional information to the existing literature that there is an alternative option for dialysis units to diminish clot burden until a more permanent solution is established through angioplasty. In one of our cases described (case 2), the fistula was deemed unsalvageable during angioplasty with large residual clot in the venous aneurysm on ultrasound. Repeated treatments with tPA infusions on dialysis resulted in objective evidence of decrease in clot burden and continued use of the same fistula for dialysis.

An obvious limitation is the small number of patients described, but we were able to demonstrate similar results in subsequent cases. It would be difficult to state with 100% confidence that resolution of clot burden was because of the tPA infusion rather than time, but existing clot is unlikely to improve by itself in such a short time period. We were able to demonstrate safety during and after infusion of tPA in the dialysis unit with no clinical evident complications.

## Conclusion

We have demonstrated that low dose tPA infusion can be used safely and effectively by vascular access nurses in the dialysis facility. It is important to note that this procedure is intended as a temporary measure until a more definitive intervention is performed or as a means of decreasing clot burden post intervention.

## Abbreviations

VA: Vascular access
HD: Hemodialysis
AVF: Arteriovenous fistula
tPA: Tissue plasminogen activator
ESRD: End stage renal disease
RC: Radio-cephalic
BC: Brachio-cephalic
